# 1D Nanostructure-Based Piezo-Generators

**DOI:** 10.3390/nano9101474

**Published:** 2019-10-17

**Authors:** Noelle Gogneau

**Affiliations:** Center for Nanosciences and Nanotechnologies—CNRS-UMR9001, Paris-Sud University, Paris-Saclay University, 10 Boulevard Thomas Gobert, F-91120 Palaiseau, France; noelle.gogneau@c2n.upsaclay.fr

With the amount of connected objects constantly on the rise, both in our daily life and in high-technology applications, it becomes critical to deal with their associated increase in energy consumption. Their energetic autonomy is currently a key worldwide challenge with strong economic and environmental benefits. The recent miniaturization of electronic micro-devices have resulted in the reduction of energy consumption to mW and even µW, combined with the progress in micro-nano-fabrication, and have opened, in these last years, new perspectives to develop autonomous power systems based on the renewable energy harvesting.

The energy harvesting of mechanical deformations and vibrations, using piezoelectric materials, is today considered as a promising way to supply nomad microelectronic devices, such as environmental or biomedical devices, portable multimedia, distributed sensor networks, or mobile communication.

One-dimensional (1D)-nanostructures, such as nanowires (NWs), nanorods (NRs), nanofibers, have recently emerged as excellent candidates in fabricating novel, ultra-compact, and high-efficient piezoelectric generators. Due to their specific properties [1,2,3,4,5,6], namely their quasi-lattice perfection (absence of dislocations), nanoscale dimensions, and large surface-to-volume ratio, these nanostructures present, comparison with their two-dimensional (2D)-film counterparts and conventional bulk materials, undeniable advantages to significantly enhance the conversion efficiency of the generators.

Since the first demonstration of electrical energy generation from ZnO NWs in 2006 [7], the piezoelectric response of 1D-nanostructures and the development of 1D-nanostructures-based Piezo-Nano-generators, have become hot topics in nanoscience.

This special issue of Nanomaterials, entitled “1D Nanostructure-Based Piezo-Generators” compiles one review paper and a series of original articles providing new insights in the field of piezoelectric energy generation with 1D-nanostructures; from studies of the piezoelectric properties of individual nanostructures to the development of macroscopic energy harvester systems.

Firstly, the review entitled “1D Piezoelectric Material Based Nanogenerators: Methods, Materials and Property Optimization” by X. Li [8] gives a clear overview of the 1D piezoelectric nanostructures with an emphasize on the characterization and optimization of their piezoelectric properties. In this study, the piezoelectric coefficients, performance of single nanostructure-based nano-piezo-generators, and structure-dependent electromechanical properties for semiconducting NWs with wurtzite and/or zinc blend phases, perovskite NWs and 1D polymers are discussed, as well as the strategies used in the improvement of their performances. This review is a good introduction in addressing the original papers published in this special issue.

Investigating the fabrication process of 1D-nanostructures, or the influence of the NW shape or heterostructuration on their piezoelectric properties, are crucial studies at the base of the development of new generator devises, with high output potential. In this way, S. Hyun et al. [9] investigated the synthesis of flexible lead-free piezoelectric nanofibers, based on BNT-ST (0.78Bi_0.5_Na_0.5_TiO_3_-0.22SrTiO_3_) ceramic and PVDF-TrFE copolymers by electrospinning. The authors evidence a correlation between the enhancement of the piezoelectric response of the nanofibers and their degree of alignment. In a second paper, J. Cardoso et al. [10] simulate the influence of various ZnO NW shapes (NWs under the brunched, non-branched and nanomushroom form) on their output potential as a function of the applied force direction. Important relations between form, geometric parameters, and output voltage were extracted. Thus, the authors demonstrate that the forces applied in the z-direction always lead to higher piezoelectric potential in non-branched and nano-mushroom structures, while the applied forces along the *x*-direction are more efficient for brunched NWs. Finally, N. Jegenyes et al. [11] have demonstrated the enhancement of output voltage generation by incorporating thick In-rich InGaN insertion in the volume of GaN NWs. The thickness and the localization of the insertion, as well as its influence on the Schottky diode, through which are harvested the piezo-generated energies, are discussed. 

One of the best methods to valorize the output potential improvement resulting from the optimization of nanostructure characteristics pass through the fabrication and testing of piezo-nano-generator devices. The paper published by Johar et al. [12] presents a flexible piezo-nano-generator integrating GaN NWs. The device performances are investigated as a function of the actuation frequency and along the time to study its long-term stability.

Finally, the physical phenomena governing the piezoelectric conversion in generators are the same in force sensor devices. The improvement of the electromechanical coupling of the 1D-nanostructures is benefits for the both applications. The paper entitled, “Piezo-Potential Generation in Capacitive Flexible Sensors Based on GaN Horizontal Wires” by Kacimi et al. [13] highlights with simulations and experimental measurements that GaN NWs, with high piezoelectric properties, can also be very useful for developing high-sensitive piezoelectric sensors.

The present special issue cannot fully reflect the complete topic of 1D-nanostructure-based piezoelectric generators. However, I am confident that, with a focus on the influence of NW characteristics on their piezoelectric properties, this special issue contributes to the research in the field and opens new perspectives in improving the electromechanical conversion efficiency, both for piezoelectric generator and piezoelectric sensor devices.
